# Preferences for Updates on General Research Results: A Survey of Participants in Genomic Research from Two Institutions

**DOI:** 10.3390/jpm11050399

**Published:** 2021-05-11

**Authors:** Casey Overby Taylor, Natalie Flaks Manov, Katherine D. Crew, Chunhua Weng, John J. Connolly, Christopher G. Chute, Daniel E. Ford, Harold Lehmann, Alanna Kulchak Rahm, Iftikhar J. Kullo, Pedro J. Caraballo, Ingrid A. Holm, Debra Mathews

**Affiliations:** 1 Department of Medicine, Department of Biomedical Engineering, and The Institute for Computational Medicine, Johns Hopkins University, Baltimore, MD 21205, USA; 2 Department of Medicine, Johns Hopkins University, Baltimore, MD 21205, USA; nflaksm1@jhu.edu (N.F.M.); dford@jhmi.edu (D.E.F.); 3 Department of Medicine and Epidemiology, Columbia University, New York, NY 10032, USA; kd59@cumc.columbia.edu; 4 Department of Biomedical Informatics, Columbia University, New York, NY 10032, USA; cw2384@cumc.columbia.edu; 5 Center for Applied Genomics, Children’s Hospital of Philadelphia, Philadelphia, PA 19104, USA; ConnollyJ1@email.chop.edu; 6 Schools of Medicine, Public Health, and Nursing, Johns Hopkins University, Baltimore, MD 21205, USA; chute@jhu.edu; 7 Department of Medicine, Division of Health Sciences Informatics, Johns Hopkins University, Baltimore, MD 21205, USA; lehmann@jhmi.edu; 8 Genomic Medicine Institute, Geisinger, Danville, PA 17822, USA; akrahm@geisinger.edu; 9 Department of Cardiovascular Medicine, Mayo Clinic, Rochester, MN 55905, USA; kullo.iftikhar@mayo.edu; 10 Department of Medicine, Mayo Clinic, Rochester, MN 55905, USA; Caraballo.Pedro@mayo.edu; 11 Division of Genetics and Genomics, Boston Children’s Hospital and Department of Pediatrics, Harvard Medical School, Boston, MA 02115, USA; ingrid.holm@childrens.harvard.edu; 12 Johns Hopkins University Berman Institute of Bioethics, Baltimore, MD 21205, USA; dmathews@jhu.edu

**Keywords:** study participation, communication, survey, general research results

## Abstract

There is a need for multimodal strategies to keep research participants informed about study results. Our aim was to characterize preferences of genomic research participants from two institutions along four dimensions of general research result updates: content, timing, mechanism, and frequency. Methods: We conducted a web-based cross-sectional survey that was administered from 25 June 2018 to 5 December 2018. Results: 397 participants completed the survey, most of whom (96%) expressed a desire to receive research updates. Preferences with high endorsement included: update content (brief descriptions of major findings, descriptions of purpose and goals, and educational material); update timing (when the research is completed, when findings are reviewed, when findings are published, and when the study status changes); update mechanism (email with updates, and email newsletter); and update frequency (every three months). Hierarchical cluster analyses based on the four update preferences identified four profiles of participants with similar preference patterns. Very few participants in the largest profile were comfortable with budgeting less money for research activities so that researchers have money to set up services to send research result updates to study participants. Conclusion: Future studies may benefit from exploring preferences for research result updates, as we have in our study. In addition, this work provides evidence of a need for funders to incentivize researchers to communicate results to participants.

## 1. Introduction

Recruiting and retaining participants for biobanks and observational studies are well-known challenges for biomedical research [1,2]. Population biobanks are essential structures to store and manage biological samples and information that can be used for research [3,4]. With the willingness to participate in biobanks correlated to opportunities to be updated about the biobanks [5], soliciting preferences will be key to maintaining successful and patient-centered population biobanks. Providing such opportunities for genomic research participants to be updated on general research results, in particular, holds promise to encourage new and continued participation [6,7] and also offers potential value back to the participant as a form of reciprocity and a signal of respect [8]. Research participants, however, have different preferences for when and how they would like to be updated [9]. Thus, there is a need to understand if there are distinct groups of individuals who have similar preferences for being updated about research (i.e., preference profiles). Such knowledge of preference profiles for target research populations can help inform what options researchers provide to eligible participants at the time of study enrollment to be inclusive. The aim of this project was to characterize the preference profiles of genomic study participants from two institutions.

There is broad recognition of a need for mechanisms for researchers to share results with participants [10]. Previous research to understand study participants’ preferences for research results have focused on three main areas: individual results, aggregate results, and general research results [11]. Individual results provide study participants with access to their own data, which may include lab measurements, genome sequences, responses to survey questions, etc. Aggregate results provide similar data types at an aggregate level. General research results include basic information about a study and its outcomes [12]. Helping participants to understand their individual results is considered a best practice and is supported in the literature [13,14,15]; however, many researchers are concerned about the feasibility of returning those data [16]. As highlighted in the National Academies of Sciences, Engineering and Medicine guidance for a new research paradigm [17], there is a balance between the value and feasibility of returning results, with justification for return being strongest when both are high. General research results may be considered the most feasible of the three types of results to return. The value of such results to study participants is similar to the value recognized with the return of aggregate results: affirming the value of their participation, building trust in the research enterprise, and education about the research process [18]. Thus, as it becomes more feasible to return individual results, the return of general research results will remain important.

There remain gaps in our knowledge of study participant preferences for the dissemination of general research results [19,20]. For biobanks, there is the capacity to generate genetic data that may have health implications for participants, raising the need to address return of individual results, aggregate results, and general research results.

Our study considers participant preferences for general research result updates along four dimensions: content, timing, mechanism, and frequency. We assessed: the level of endorsement of a preference statement and ranked those statements along the four dimensions; identified profiles of individuals with similar preferences; and examined associations between preference profiles with opinions about using clinical information in research and comfort with reallocating money for research activities to set up services providing research result updates to participants.

## 2. Materials and Methods

This was a web-based cross-sectional survey study at two institutions (Johns Hopkins University (JHU) and Columbia University (CU)) of adult patients who had previously enrolled in a research study. The survey was administered from 25 July 2018 to 5 December 2018.

### 2.1. Recruitment Criteria and Survey Distribution

At JHU, we recruited patients who were seen as inpatients or outpatients at Johns Hopkins Hospital, participated in one of 35 studies registered with the database of Genotypes and Phenotypes (dbGAP, https://www.ncbi.nlm.nih.gov/gap/(accessed on May 7, 2021), and had a MyChart (patient portal) account they had logged into within the last 12 months. Patients were excluded if they were known to be deceased, had previously opted out of being contacted for recruitment through MyChart, had an invalid or null email address, or were previously contacted as part of a related pilot survey study. For CU, we recruited patients who were recently seen in outpatient clinics at Columbia/New York-Presbyterian Hospital (including the Herbert Irving Comprehensive Cancer Center), and had consented to be re-contacted by email for research. Surveys were distributed using a Web-based Qualtrics survey embedded in an email distributed by MyChart (at JHU) and by the site PI (at CU).

### 2.2. Measures

Our primary outcome was the preference of a participant for general research results, along the four dimensions mentioned earlier, with potential preference modifiers based on social and demographic characteristics.

#### 2.2.1. Social and Demographic Characteristics

Demographic measures included gender, age, ethnicity, race, and highest level of education. We also asked respondents to report their primary health care institution, if they speak English as their first language, and if they remembered donating samples of any kind for use in research. We also asked respondents if they wanted to be updated about general research results. Respondents were asked if they agree or disagree with three statements about desired types of updates: research on health topics I choose, research that uses samples and clinical information from my institution, research that uses my samples and clinical information (Questions 6–8). Response options were on a 3-point Likert scale (agree, neither agree nor disagree, disagree). Taking an opt-in perspective, we labeled an individual as “want to be updated” if they answered “agree” to at least one of Questions 6–8. Otherwise they were labeled as “do not want/no preference to be updated.” See Supplementary Materials for survey.

#### 2.2.2. Preferences for Research Updates

Update content: Respondents were asked about their preference for each of seven types of content updates: number of published articles about the research, brief descriptions of the research, brief descriptions of major findings from the research, brief descriptions of any media coverage of the research, educational material about the research, community events about the research, and announcements about online platforms to interact with others with similar interests (Questions 10–16). Response options were on a 3-point Likert scale (high, medium, low).

Update timing: Respondents were asked about their preference for each of seven options for when to receive updates: when the research is completed, when research findings are reviewed (validated) by other researchers and clinicians, when research findings are published, when educational materials about the research are available, when there is a media release about the research, when there is a community event about the research, and when status of the research changes (Questions 17–23). Response options were on a 3-point Likert scale (high, medium, low).

Update mechanism: Respondents were asked about their preference for each of five mechanisms to receive updates: a call on your phone to deliver a prerecorded message, a text (SMS) message, a mailed newsletter, an email, and an electronic newsletter by email (Questions 26–30). Response options were on a 3-point Likert scale (high, medium, low).

Update frequency: Respondents were asked how often they would like to receive updates about the research (Question 25): never, less than once a year, once a year, quarterly (once every 3 months), once a month, once every 2 weeks, once a week, and more than once a week. We created a three-group measure to represent a preference for update frequency: once a month or more frequent (once a month, once every 2 weeks, once a week, more than once a week); once every 3 months (quarterly); and once a year or less frequent (never, less than once a year, once a year).

#### 2.2.3. Opinions about Research Focus and Budgeting

Interest in research focus: Respondents were asked if it is important that their samples and clinical information are used in different types of research: a disease in general, a disease that effects a loved one, and diseases seen in their community (Questions 3–5). Response options were on a 3-point Likert scale (agree, neither agree nor disagree, disagree). An individual was labeled as interested in research focus if they answered “agree” to at least one of Questions 3–5. Otherwise they were labeled as no interest in/indifferent on research focus.

Comfort with budgeting less money for research: Respondents were asked if they would support budgeting a bit less money for research activities so that researchers have money to set up services to send research study updates to study participants (Question 31). Response options were yes, no, unsure. An individual was labeled as comfortable with less money for research if they answered yes to Question 31, and labeled as not comfortable/unsure if they answered no or unsure to Question 31.

### 2.3. Analytical Strategy

Descriptive analyses were used for social and demographic characteristics and research update preferences.

We assessed the level of endorsement of a preference statement and ranked preference statements by ordering the frequency of individuals indicating that they agree with a statement from the largest (rank 1) to the smallest. We hypothesized that preference statements with high endorsement (>50% of the survey respondents) would be content types that are already routinely prepared by research teams (e.g., description of study purpose and goals), that are provided by research teams at common times points (e.g., when the research is completed), that are digitally-based (e.g., email or SMS texting updates), and are at a frequency of once a year or more (e.g., once every three months, once a month or more frequent).

We tested our hypothesis that there would be distinct preference profiles among surveyed individuals by conducting a hierarchical cluster analysis based on the four dimensions of general research result updates: content, timing, mechanism and frequency. A cluster dendrogram diagram was created to show a hierarchical clustering relationship between similar sets of data. In order to further characterize preference profiles, comparisons between clusters were made using χ^2^ test. To test our hypotheses that preference profiles would be associated with different opinions about how clinical information is used in research, we conducted a bivariate analysis by χ^2^ test. We also tested associations with different demographics, also using χ^2^ test. All statistical analyses were conducted using R (version 3.6.2).

## 3. Results

### 3.1. Social and Demographic Characteristics

A total of 397 participants completed the survey. Almost two thirds of the survey participants were female (268, 68%), more than two thirds were 45 years and older (290, 73%), nearly a third of participants had a bachelor’s degree (121, 31%) and 44% of participants had a graduate or professional degree. The majority were non-Hispanic (362, 91%), with 84.6% non-Hispanic White. Most of the participants were JHU patients (313, 79%), with the remaining from CU. Most of the participants (382, 96%) wanted to be updated about the research (Table 1).

### 3.2. Ranking of Preferences for Research Updates

**Figure 1:**
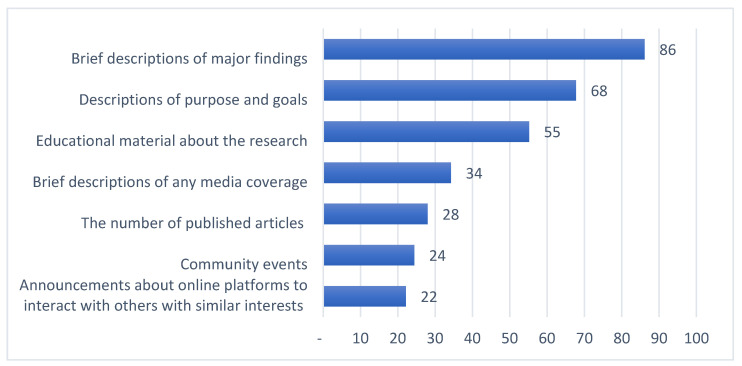
Percent of survey respondents indicating that they agree with each update content statement.

**Figure 2:**
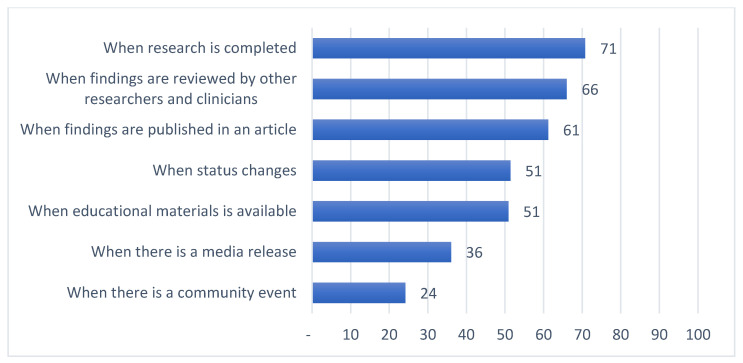
Percent of survey respondents indicating that they agree with each update timing statement.

**Figure 3:**
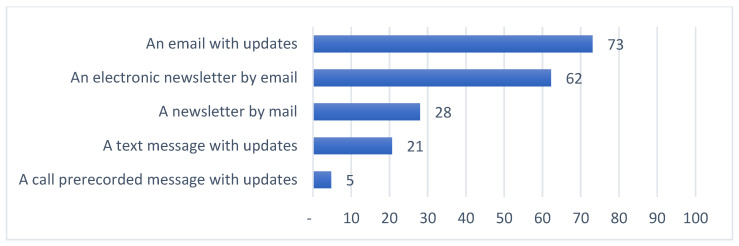
Percent of survey respondents indicating that they agree with each update mechanism statement.

**Figure 4:**
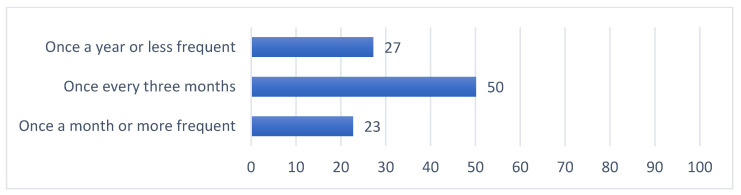
Percent of survey respondents indicating that they agree with each update frequency statement.

Summaries of preferences for updates on general research results along four dimensions are provided in Figure 1, Figure 2, Figure 3 and Figure 4. Among those preferences receiving a high level of endorsement (>50% of the survey respondents), the highest-ranked content type was brief descriptions of major findings, followed by descriptions of purpose and goals, and educational material about the research (Figure 1); the highest-ranked update timing was when the research is completed, followed by when findings are reviewed by other researchers and clinicians, when findings are published, and when the status of the study changes (Figure 2); the highest-ranked update mechanism was via email, followed by an electronic newsletter by email (Figure 3); and the highest-ranked update frequency was every three months (Figure 4).

### 3.3. Cluster Analyses to Identify Preference Profiles

Our cluster analysis identified four preference profiles:

1. cluster 1 (*n* = 75), moderate value-driven and moderate engagement-driven, MVME
2. cluster 2 (*n* = 170), moderate value-driven and low engagement-driven, MVLE
3. cluster 3 (*n* = 69), low value-driven and low engagement-driven, LVLE
4. cluster 4 (*n* = 83), high value-driven and high engagement-driven, HVHE

The strength of endorsement with content type, timing, mechanism and frequency preference dimension attributes define each preference profile (Table 2). The rankings within each preference dimension can help to differentiate the four preference profiles. Most of the distinguishing preferences were in the content type and timing categories. For instance, receiving brief descriptions of any media coverage and receiving updates when there is a media release were top preferences for cluster 4, but those preferences were ranked below 3 for all other clusters. In addition, receiving the number of published articles was the lowest ranked content type (ranked 7) for clusters 3 and 4, but was ranked 4 for both clusters 1 and 2.

Other distinguishing preferences between clusters for timing were to receive updates when the status of the study changes (ranked differently for all clusters) and to receive updates when there is a community event (ranked high for only cluster 4). The preference to receive updates when the status of the study changes was ranked in the top 3 for clusters 1 and 3 but not for clusters 2 and 4 (ranked 5 and 4). Receiving updates when there is a community event was ranked lowest for all but cluster 4 (ranked 2). Finally, the highest-ranked preference for update frequency was every three months for all clusters except cluster 1 (ranked 2). For cluster 1, the highest-ranked preference for update frequency was once a month or more.

To label clusters, we considered distinguishing preference dimension attributes. The media coverage and published articles content types conveyed the value of the research, and were used to label clusters as low, medium or high value-driven. We also considered update timing, update frequency, and two update content types (community events and announcements about online platforms to interact with others) that conveyed engagement to label clusters as low, medium or high engagement-driven.

### 3.4. Opinions about Research Focus and Budgeting among Preference Profiles

There was a statistically significant difference between preference profiles by comfort with budgeting less money for research activities so that researchers have money to set up services to send research updates to participants (Table 3): In cluster 2, 13.5% (23/170) of respondents agreed with this statement vs. 21.7–31.3% of respondents from the three other clusters. There was no statistically significant difference in preference profile by interest in research focus.

### 3.5. Characteristics of Preference Profiles

The demographic characteristics of the four preference profiles are shown in Table 4. There were no statistically significant differences in preferences by gender, age group or education among the clusters. Additionally, there was a borderline significance in preference profiles between the two institutions (*p* = 0.047). Differences by demographic characteristics are summarized in Supplemental Materials Table S1.

## 4. Discussion

In this study, we explored preferences for updates on general research results, including the content, timing, mechanism and frequency, among individuals who have previously donated samples and clinical information for use in genomic research (Figure 1, Figure 2, Figure 3 and Figure 4, Table 2). This work confirms the findings in the literature indicating that most research participants want results from studies in which they participate [19,21,22,23,24,25,26]. A “one-size-fits-all” dissemination approach, however, is not sufficient to address participant desires, because we found at least four clusters of preference profiles. In our assessments of specific preferences receiving high endorsement, our findings were mixed with respect to our hypotheses.

First, as hypothesized, we found that there was high endorsement of preferences to receive updates on content types that are already routinely prepared by research teams, including preparing descriptions of study purpose and goals and brief descriptions of major findings. Most clusters also showed a high endorsement for updates on both study purpose and goals (clusters 1, 2, and 4), and on brief descriptions of major findings (clusters 1, 2, and 4). With some revisions to target a lay public audience, those descriptions may be repurposed to provide to participants at a low cost to the study team. There was also, however, high endorsement of preferences to receive updates on one content type that is less often prepared by research teams: educational material about the research. Two clusters showed a high endorsement for updates on educational material (clusters 1 and 4). The desire for educational material about the research has been described in one prior study where participants wanted to know how research findings apply to health care and policy and what impact it has for future decision-making in healthcare [19].

Second, in support of our hypothesis that there would be a preference for updates at time points that are already common for research studies, we found that there was high endorsement of preferences to receive updates when the research is completed. Most clusters also showed a high endorsement for updates when the research is completed (clusters 1, 2, and 4). For some forms of research, such as community-based research, it is already considered best practice for researchers to disseminate updates when the research is completed [19,26]. Less common time points for which there was also high endorsement included: when findings are reviewed by other researchers and clinicians, when findings are published, and when the status of the study changes. Three clusters showed a high endorsement for updates when findings are reviewed by others (clusters 1, 2, and 4); three for when findings are published (clusters 1, 2, and 4); and two for when the status of a study changes (clusters 1 and 4). The desire to be updated when findings are reviewed by other researchers and clinicians, and when findings are published, however, is consistent with the work of others that indicates study participants are willing to wait until results have been reviewed by other researchers for accuracy and until after the study has been published [24].

Third, as we hypothesized, our review of preferences for mechanisms to deliver updates indicated high endorsement of digital approaches: email with updates and electronic newsletter by email. Three clusters also showed high endorsement for email (clusters 1, 2, and 4), and for electronic newsletter by email (clusters 1, 2, and 4). Texting (SMS) updates, however, were not included in this group and none of the clusters showed high endorsement. Given that enabling mechanisms for text message updates may be more expensive than sending emails, this result adds to the literature showing that participants are open to receiving results through low-cost digital channels such as email and websites [23,24].

Fourth, there was high endorsement of preferences to receive updates every three months. Two clusters also showed high endorsement (clusters 2 and 4). This finding was complementary to results from a focus group study where participants preferred multiple contacts over time (at least every three months) to be kept informed [19]. While studies registered with ClinicalTrials.gov must report updates when the recruitment status changes (e.g., ongoing, completed, terminated), it is not required that these updates trigger communications with study participants. These findings highlight content types and mechanisms that research teams do not typically use, but that could be prioritized when designing research dissemination strategies.

In addition to finding several commonly endorsed preferences among clusters, we also identified several unique characteristics (Table 2 and Table 3). The MVME group (cluster 1) was distinct from other clusters as the only one with a majority of survey respondents indicating a preference for updates once a month or more frequent, indicating a possible greater desire to stay informed than other groups. The largest preference profile (cluster 2-MVLE) indicated that few wanted to take money away from research (14%, 23/170) and few endorsed more frequent updates (1%, 1/170, endorsing a preference for updates once a month or more frequent). The other three preference profiles included more individuals that felt comfortable with budgeting less money for research (20% to 33%) and that endorsed a preference for updates once a month or more frequent (17% to 74%). The smallest preference profile (cluster 3-LVLE) showed lower ranging endorsement of preferences in all four dimensions (<50% across all dimensions). Distinct for cluster 4 (HVHE) was that a majority endorsed a preference for updates when there is a media release about the research (92%, 76/83) when compared to other clusters (7–33%).

Finally, we tested associations of preference profiles with participant characteristics and with opinions about research focus and about providing funding to update study participants (Table 3 and Table 4). Unlike the findings of others, showing that preferences vary with study topic and participant characteristics [23,25], we did not find differences in opinions about research focus or demographic characteristics between the preference profiles. Our finding that there are statistically significant differences between preference profiles with respect to comfort budgeting less money for research, suggests an opportunity for funders to incentivize researchers to communicate results to participants, for example, by requiring and providing funding to update study participants. Without such a budget, patients seeking such feedback are likely not to participate, and so research will continue to recruit only a subset of target patient groups. Others have also encouraged funders to provide incentives for researchers, given that many now call for better dissemination of general research results [24].

### 4.1. Limitations

This study has some limitations. First, survey participants had already decided to participate in research and most of them wanted to be updated about the research. Our study population, therefore, may not represent the general public with respect to their motivations to participate in research. For example, personal/family benefit is a common motivator to participate in large-scale genomic sequencing studies [27]. For our selected studies, there were not opportunities for personal/family benefit; thus, this was unlikely to be a motivator. Second, demographic characteristics of the current study population differ from the general US population. This survey population represents an older, mostly white race, highly educated and predominantly female population. Although the study population is different from the general population, other studies have shown that the characteristics of individuals that agree to participate in health-related studies are different from the general population [28,29,30]. This may be, at least in part, due to ineffective outreach to groups that are less willing to participate. Others have found that a systematic plan to contact and track participants or potential participants may differentiate effective from ineffective interventions to recruit and retain study participants [31]. Our work helps to lay the foundation for addressing this limitation by identifying different types of update content, mechanisms, timings, and frequencies that might be considered when developing a plan for recruiting and retaining participants.

### 4.2. Implications for Stakeholders

Our cluster analysis identified four different preference profiles among survey participants, which adds to existing evidence suggesting that there exists variability in the communication preferences of study participants. There is a growing desire to attract diverse populations (with potentially diverse views on what results are valuable) to participate in initiatives such as the All of Us Research Program [20,32]. A multi-pronged approach is required to meet the needs and preferences of individuals from diverse populations. Though the range and granularity of data being collected in research is increasing, preferences with regard to the types, timing and approaches to return results to participants is largely uncharted territory [20]. Models to return general research results that are multidimensional and responsive to participant preferences hold promise to provide the most value to study participants [33].

One study, for example, found that focus group participants were open to a variety of pathways and platforms for receiving study findings [19]. Participants wanted to have control over how, when, and how often they receive study results. They also wanted the opportunity to adjust the frequency and timing during the course of a longitudinal study. Furthermore, recent studies of the return of individual results have captured experiences with participant choice for the return of genomic results, indicating that some elect different choices when offered options [34,35]. Such processes to offer options for the return of individual results might be extended to also include general research results like those explored in this study.

Our efforts and the efforts of others to characterize the desires of study participants justify the use of multimodal strategies that could be considered when disseminating research findings. To lower the potential burden of providing research result updates, biobank data management systems might provide mechanisms that automate or semi-automate the process of curating preferences and for delivering some update types. As an important step in this direction, some groups have explored IT strategies to manage dynamic consent [36,37,38,39] that might be adapted for managing preferences for and delivery of research result updates. Future studies on processes to return results may benefit from exploring preference profiles, as we have in the current study, and also using those profiles in research result dissemination strategies.

## 5. Conclusions

This study adds an in-depth exploration of the specific preferences of research participants for different types of content, mechanisms, timings, and frequencies for updates on general research results. We also identify four preference profiles among survey participants that had already decided to participate in research, which adds to existing evidence suggesting variability in the communication preferences of study participants. Future studies on processes to return a range of research result types including individual, aggregate, and general research results may benefit from exploring preference profiles as we have in our study. Furthermore, this work provides evidence of a need for funders to incentivize researchers to communicate results to participants.

## Figures and Tables

**Table 1:** Social and demographic characteristics of the survey respondents.

Variables	Categories	N (%, N = 397)
Gender	Male	120 (30.2%)
	Female	268 (67.5%)
	Prefer not to say/missing	9 (2.3%)
Age	18–29 years old	9 (2.3%)
	30–44 years old	90 (22.7%)
	45–59 years old	136 (34.3%)
	60 years old or more	154 (38.8%)
	Prefer not to say/missing	8 (2.0%)
Education (highest level)	Less than high school	1 (0.3%)
	High school graduate or GED	26 (6.5%)
	Some college	67 (16.9%)
	Bachelor’s degree	121 (30.5%)
	Graduate or professional degree	174 (43.8%)
	Prefer not to say/missing	8 (2.0%)
Ethnicity	Hispanic or Latino	25 (6.3%)
	Non-Hispanic	362 (91.2%)
	Prefer not to say/missing	10 (2.5%)
English (first language)	Yes	368 (92.7%)
	No	21 (5.3%)
	Prefer not to say/missing	8 (2.0%)
Race		
	White Caucasian	336 (84.6%)
	Black African American	27 (6.8%)
	Asian or Asian American	5 (1.3%)
	Multiracial	19(4.8%)
	Other/missing	10 (2.5%)
Primary healthcare institution		
	Johns Hopkins University Columbia University	313(78.8%) 84(21.2%)
Remember donating sample		
	Yes	327(82.4%)
	No	38(9.6%)
	Unsure	32(8.1%)
Want to be updated about research	Yes	382(96.2%)
	No/Unsure	15(3.8%)

**Table 2:** Percent of survey respondents endorsing a preference statement in each cluster and its ranking within each update dimension (content, timing, mechanism, and frequency).

Research Updates Preferences	Clusters					Rank of Statements			
	1	2	3	4	*p*	1	2	3	4
	N = 75	N = 170	N = 69	N = 83					
**Update Content**									
Brief descriptions of major findings	74 (98.7%)	163 (95.9%)	28 (40.6%)	77 (92.8%)	<0.001	1	1	1	1
Descriptions of purpose and goals	66 (88.0%)	132 (77.6%)	4 (5.8%)	67 (80.7%)	<0.001	2	2	3	3
Educational material about the research	54 (72.0%)	84 (49.4%)	14 (20.3%)	67 (80.7%)	<0.001	3	3	2	3
The number of published articles	31 (41.3%)	38 (22.4%)	1 (1.4%)	41 (49.4%)	<0.001	4	4	7	7
Brief descriptions of any media coverage	31 (41.3%)	32 (18.8%)	2 (2.9%)	71 (85.5%)	<0.001	4	5	4	2
Community events	13 (17.3%)	17 (10.0%)	2 (2.9%)	65 (78.3%)	<0.001	7	6	4	5
Announcements about online platforms to interact with others with similar interests	18 (24.0%)	15 (8.8%)	2 (2.9%)	53 (63.9%)	<0.001	6	7	4	6
**Update Timing**									
When findings are reviewed by other researchers and clinicians	67 (89.3%)	119 (70.0%)	12 (17.4%)	64 (77.1%)	<0.001	1	1	3	7
When research is completed	67 (89.3%)	115 (67.6%)	27 (39.1%)	72 (86.7%)	<0.001	1	2	1	4
When findings are published in an article	61 (81.3%)	98 (57.6%)	11 (15.9%)	73 (88.0%)	<0.001	2	3	4	2
When educational materials are available	50 (66.7%)	74 (43.5%)	7 (10.1%)	71 (85.5%)	<0.001	4	4	5	6
When status changes	53 (70.7%)	65 (38.2%)	14 (20.3%)	72 (86.7%)	<0.001	3	5	2	4
When there is a media release	25 (33.3%)	37 (21.8%)	5 (7.2%)	76 (91.6%)	<0.001	5	6	6	1
When there is a community event	10 (13.3%)	12 (7.1%)	1 (1.4%)	73 (88.0%)	<0.001	6	7	7	2
**Update Mechanism**									
An email	61 (81.3%)	131 (77.1%)	27 (39.1%)	71 (85.5%)	<0.001	1	1	1	1
An electronic newsletter by email	48 (64.0%)	115 (67.6%)	20 (29.0%)	64 (77.1%)	<0.001	2	2	2	2
A newsletter by mail	28 (37.3%)	37 (21.8%)	11 (15.9%)	35 (42.2%)	<0.001	3	3	4	3
A text message	20 (26.7%)	22 (12.9%)	12 (17.4%)	28 (33.7%)	0.001	4	4	3	4
A call prerecorded message	5 (6.7%)	3 (1.8%)	1 (1.4%)	10 (12.0%)	0.002	5	5	5	5
**Update Frequency**									
Once every three months	19 (25.3%)	97 (57.1%)	30 (43.5%)	53 (63.9%)		2	1	1	1
Once a year or less frequent	0 (0%)	72 (42.4%)	27 (39.1%)	9 (10.8%)	<0.001	3	2	2	3
Once a month or more frequent	56 (74.7%)	1 (0.6%)	12 (17.4%)	21 (25.3%)		1	3	3	2

**Table 3:** Percent of survey respondents from each cluster indicating comfort with budgeting less money for research, and an importance to them that their samples and clinical information be used in certain types of research.

	Clusters				
Opinions	1	2	3	4	*p* Value
	N = 75	N = 170	N = 69	N = 83	
Comfortable with less money for research	18 (24.0%)	23 (13.5%)	15 (21.7%)	26 (31.3%)	0.01
Interest in research focus	63 (84.0%)	142 (83.5%)	52 (75.4%)	76 (91.6%)	0.06

**Table 4:** Percent of survey respondents assigned to each cluster, according to demographic characteristics, health institution, and opinions about budgeting less money for research activities and interest in research focus.

Characteristics		Clusters				
		1	2	3	4	*p* Value
		75	170	69	83	
Gender	Male	16 (21.3%)	55 (32.4%)	23 (33.3%)	26 (31.3%)	
	Female	57 (76.0%)	111 (65.3%)	43 (62.3%)	57 (68.7%)	0.27
Age	18–59	42 (56.0%)	107 (62.9%)	38 (55.1%)	48 (57.8%)	
	60+	31 (41.3%)	59 (34.7%)	29 (42.0%)	35 (42.2%)	0.59
Education	Bachelor’s degree or less	42 (56.0%)	82 (48.2%)	42 (60.9%)	49 (59.0%)	
	Graduate or professional degree	31 (41.3%)	84 (49.4%)	25 (36.2%)	34 (41.0%)	0.29
Health care institute	Columbia University	20 (26.7%)	27 (15.9%)	21 (30.4%)	16 (19.3%)	
	Johns Hopkins University	55 (73.3%)	143 (84.1%)	48 (69.6%)	67 (80.7%)	0.047

## Data Availability

The survey instrument used in this study is included as part of the Supplementary Materials. Data supporting Figure 1, Figure 2, Figure 3 and Figure 4, Table 1, Table 2 and Table 3 and Supplementary Table S1 can be made available upon request.

## Supplementary Materials

The following are available online at https://www.mdpi.com/article/10.3390/jpm11050399/s1, Table S1: Demographic characteristics of survey respondents, by institution. Materials S1: Survey.
