# Supplementary material for: Preferences for Updates on General Research Results: A Survey of Participants in Genomic Research from Two Institutions
Source: J Pers Med. 2021 May 11;11(5):399. doi: 10.3390/jpm11050399 (PMC8151672; doi:10.3390/jpm11050399)
Supplement: Supplementary file 1 [file jpm-11-00399-s001.zip › SupplementaryTable.pdf]

Supplemental Materials

Demographic characteristics		Total (N=397)	Columbia University (N=84)	Johns Hopkins University (N=313)	P value
Gender	Male	120 (30.2%)	34 (40.5%)	86 (27.5%)	
	Female	268 (67.5%)	48 (57.1%)	220 (70.3%)	0.03
	Prefer not to say	9 (2.3%)	2 (2.4%)	7 (2.2%)	
Age	18-44	99 (24.9%)	10 (11.9%)	89 (28.4%)	
	45-59	136 (34.3%)	22 (26.2%)	114 (36.4%)	<0.001
	60+	154 (38.8%)	51 (60.7%)	103 (32.9%)	
	Prefer not to say	8 (2.0%)	1 (1.2%)	7 (2.2%)	
Education	Bachelor's degree or less	215 (54.2%)	42 (50.0%)	173 (55.3%)	
	Graduate or professional degree	174 (43.8%)	41 (48.8%)	133 (42.5%)	0.45
	Prefer not to say	8 (2.0%)	1 (1.2%)	7 (2.2%)	
Race	White	343 (86.4%)	73 (86.9%)	270 (86.3%)	1.00
