# Supplementary material for: Preferences for Updates on General Research Results: A Survey of Participants in Genomic Research from Two Institutions
Source: J Pers Med. 2021 May 11;11(5):399. doi: 10.3390/jpm11050399 (PMC8151672; doi:10.3390/jpm11050399)
Supplement: Supplementary file 1 [file jpm-11-00399-s001.zip › Qualtrics-Survey-Software.pdf]

## Introduction

## Introduction

In the past, you or your child have donated a sample for use in research. We want to understand your opinions on how those samples and clinical information might be used and how you would like to be updated on the outcomes from using those samples.

- A ***sample*** is any kind of human material including tissue, blood, urine, skin, bone marrow, hair, and teeth. Tissue can also be the whole or a part of an organ that has been removed during surgery.
- ***Clinical information*** may include medical history, diagnoses, medications, treatment plans, immunization dates, allergies, radiology images, and laboratory and test results.

This survey will take about 15 minutes to complete. Your responses will help us understand how you might like your healthcare institution to keep you informed about what happens to donated samples.

**Protocol Number:** IRB00107475 (Johns Hopkins University)

**Your completion of this survey or questionnaire will serve as your consent to be in this research study.**

Please click "NEXT" to begin

## Institution

**1. Please select your primary health care institution**

- Children’s Hospital of Philadelphia
- Cincinnati Children’s Hospital
- Columbia University
- Geisinger
- Johns Hopkins University
- Marshfield Clinic Health System
- Mayo Clinic
- ________ Other

## Trust and opinions about using clinical information in research

**Some people choose to donate sample(s) for use in research. A sample is any kind of human material including tissue, blood, urine, skin, bone marrow, hair, teeth. A sample can also be the whole or a part of an organ that has been removed during a surgery.**

**2. Do you remember donating samples of any kind for use in research?**

- Yes
- No
- Unsure

**Please show if you agree or disagree with the following statements:**

| | *Agree* | *Neither agree nor disagree* | *Disagree* |
|---|---|---|---|
| 3. It is important that my samples and clinical information be used in research on a disease I have. | ○ | ○ | ○ |
| 4. It is important that my samples and clinical information be used in research on a disease that affects a loved one. | ○ | ○ | ○ |
| 5. It is important that my samples and clinical information be used in research on diseases seen in my community | ○ | ○ | ○ |

## Desire for updates

**Please show if you agree or disagree with the following statements:**

| | *Agree* | *Neither agree nor disagree* | *Disagree* |
|---|---|---|---|
| 6. I would want to be updated about research on health topics I choose (e.g., diabetes, asthma, mental illness) | ○ | ○ | ○ |
| 7. I would want to be updated about research that use samples and clinical information from [Primary Health care Institution from Question 1]_____ | ○ | ○ | ○ |
| 8. I would want to be updated about research that use my samples and clinical information. | ○ | ○ | ○ |

9. **Are there any updates about research you would not want? (Select all that apply)**

- ☐ I would not want updates from institutions that are not [Primary Institution from Question 1].
- ☐ I would not want updates about research that do not use my samples and clinical information.

☐ I would not want updates about research that is not on health topics I choose (e.g., diabetes, asthma, mental illness).

### Preferences - types of updates

**Researchers often choose to provide updates to individuals after they donate samples and clinical information for research.**

**For research that you decide to follow, what types of updates do you think you would want?**

***Please rate your preference (high, medium, or low)***

| | High | Medium | Low |
|---|---|---|---|
| 10. The number of published articles about the research | ○ | ○ | ○ |
| 11. Brief descriptions of the research (e.g., purpose and goals) | ○ | ○ | ○ |
| 12. Brief descriptions of major findings from the research | ○ | ○ | ○ |
| 13. Brief descriptions of any media coverage (e.g., magazine, local news) about the research | ○ | ○ | ○ |
| 14. Educational material about the research (e.g., symptoms to recognize diabetes, asthma, mental illness) | ○ | ○ | ○ |
| 15. Community events about the research | ○ | ○ | ○ |
| 16. Announcements about online platforms to interact with others with similar interests (e.g., Facebook groups, discussion boards) | ○ | ○ | ○ |

## Preferences - frequency of updates

**For research you decide to follow, when do you think you would want to get updates?**

***Please rate your preference (high, medium, or low)***

| | High | Medium | Low |
|---|---|---|---|
| 17. When the research is completed | ○ | ○ | ○ |
| 18. When findings from the research are reviewed (validated) by other researchers and clinicians | ○ | ○ | ○ |
| 19. When findings from the research are published in an article | ○ | ○ | ○ |
| 20. When educational materials about the research is available | ○ | ○ | ○ |
| 21. When there is a media release (e.g., magazine, local news) about the research | ○ | ○ | ○ |
| 22. When there is a community event about the research | ○ | ○ | ○ |
| 23. When status of the research changes (e.g., recruiting, active, terminated, completed) | ○ | ○ | ○ |
| 24. At a frequency I specify (e.g., once a year, once a month) | ○ | ○ | ○ |

**25. How often do you think you would like to get updates about research conducted on your donated sample and clinical information that you decide to follow?**

○ Never

- Less than once a year
- Once a year
- Quarterly (once every 3 months)
- Once a month
- Once every 2 weeks
- Once a week
- More than once a week

## Preferences - mechanisms to send updates

**For research you decide to follow, how do you think you would want to be contacted about updates?**

***Please rate your preference (high, medium, or low)***

| | High | Medium | Low |
|---|---|---|---|
| 26. A call on your phone to deliver a prerecorded message with updates | ○ | ○ | ○ |
| 27. A text message with updates | ○ | ○ | ○ |
| 28. A newsletter by mail | ○ | ○ | ○ |
| 29. An email with updates | ○ | ○ | ○ |
| 30. An electronic newsletter by email | ○ | ○ | ○ |

## Costs of providing updates and engagement activities

**31. Researchers have limited funds to support research activities. Setting up services to send updates on research (such as through prerecorded messages) will cost money that may have to come out of the research budget.**

**Do you support budgeting a bit less money for research activities so that we have money to set up services?**

- ○ Yes
- ○ No
- ○ Unsure

## Demographics

**Please answer the following questions about your background:**

**32. What is your gender?**

- ○ Male
- ○ Female
- ○ Other
- ○ Prefer not to say

**33. Do you speak English as your first language?**

- ○ Yes
- ○ No
- ○ Prefer not to say

**34. What is your ethnicity?**

- ○ Hispanic or Latino
- ○ Non-Hispanic

**35. What is your race?**

- ☐ White or Caucasian
- ☐ Black or African American
- ☐ American Indian or Alaska Native
- ☐ Hawaiian or Pacific Islandar
- ☐ Asian or Asian American
- ☐ ________ Other

**36. What is your age group?**

- ○ 18-29 years old
- ○ 30-44 years old
- ○ 45-59 years old
- ○ 60 years old or more

**37. What is your highest level of education?**

- ○ Less than high school
- ○ High school graduate or GED
- ○ Some college
- ○ Bachelor's degree
- ○ Graduate or professional degree

Please click "NEXT" to complete this survey.
